# Natural Occurrence of Mycotoxins in Maize in North China

**DOI:** 10.3390/toxins14080521

**Published:** 2022-07-29

**Authors:** Sirui Cheng, Xiaoxiao Feng, Guoxin Liu, Nan Zhao, Jing Liu, Zhimeng Zhang, Nan Yang, Luqi Zhou, Minhao Pang, Bowen Tang, Jingao Dong, Bin Zhao, Yingchao Liu

**Affiliations:** State Key Laboratory of North China Crop Improvement and Regulation, College of Plant Protection, Hebei Agricultural University, Baoding 071001, China; chengsirui0424@126.com (S.C.); fengxiaoxiao@hebau.edu.cn (X.F.); liuguoxin0374@163.com (G.L.); z18034217898@163.com (N.Z.); liujing10192022@163.com (J.L.); vanilla2022@126.com (Z.Z.); y18330211790@163.com (N.Y.); luqizhou_hbnd@126.com (L.Z.); pangminhao@hebau.edu.cn (M.P.); tangbowen1992@163.com (B.T.)

**Keywords:** mycotoxins, occurrence, maize

## Abstract

Mycotoxins seriously threaten the quality of maize seriously around the world. A total of 426 samples of maize kernel from northeast and northwest China were analyzed in this study. Ultra-performance liquid chromatography–tandem mass spectrometry (UPLC–MS/MS) was performed to analyze the mycotoxin contamination of maize samples. The results showed that it was contaminated by mycotoxins in maize. The average contamination levels of fumonisins, deoxynivalenol, aflatoxins, zearalenone, ochratoxin A, T-2 and HT-2 were 937, 431, 22, 27, 2 and 12 μg/kg, respectively. Concentration of mycotoxins in some samples exceeded their limit, but most were still at safe levels. The contamination level of FBs and DON were most significative. The proportion of mycotoxins exceeding the maximum limit standard was in the following order: 8.0%, 8.0%, 7.0%, 1.6%, 1.4% and 0.0%. The contamination of mycotoxins in maize varies from region to region.

## 1. Introduction

Animal feed and agricultural commodities can be affected by mycotoxins, which are low molecular weight secondary metabolites produced by fungi [1]. Mycotoxins in crops and plant products have the characteristics of diverse sources, strong stability and posing serious health hazards. The most widely studied mycotoxins are fumonisins (FBs), aflatoxin (AFB), ochratoxin A (OTA), deoxynivalenol (DON) and zearalenone (ZEN). FBs was a kind of water-soluble secondary metabolites mainly produced by *Fusarium verticillioides* and *F. proliferatum* [2,3]. FBs are a family of toxins, of which FB_1_ accounts for more than 70% and has a high toxicity and contamination level. FBs can cause equine leukoencephalomalacia, pig pulmonary edemaesophageal cancer and so on [4]. AFBs have commonly contaminated maize and other types of crops during production, harvest and storage, and have been considered the most important mycotoxins due to their hepatotoxicity and carcinogenicity [5]. Mycotoxins can adversely affect many organs and systems, including the liver, kidneys, immune, reproduction and developmental systems, and can also cause cancer due to their toxic and carcinogenic properties [6,7]. Mycotoxins increase their toxicity through synergy with other toxins [8]. To protect consumer health, the China Food and Drug Administration and the General Administration of Quality Supervision, Inspection and Quarantine revised the limits of mycotoxins in food and feed in 2017. The US Food and Drug Administration (FDA) and European Commission also have set their limits for mycotoxins.

Currently, maize has the largest acreage in the world. Maize ear rot is more seriously contaminated and is infected by a variety of pathogens, including *Fusarium* Spp., *Aspergillus* Spp., *Penicillium* Spp. and *Trichoderma* Spp., among which fusarium is the main pathogenic microorganism [9], which not only leads to the decrease in maize yield and quality but also produces mycotoxins that affect the health of animal and human. The northeast and northwest regions are the two major maize-producing areas in China. Therefore, the maize yield and safety of these two districts are very important to China. Mycotoxins in cereals are extensively monitored in countries around the world. The average concentration of FBs in maize kernel samples from eight provinces of China in 2014 was 817 μg/kg, with the positive rate of 67.1% [10]. Maize, the main staple food in sub-Sahara Africa, was also contaminated with mycotoxins to varying degrees [11]. Mycotoxins such as FBs, ZEA and AFs were analyzed in some samples of Spanish maize kernels that exceeded the EU limit standards [12]. Mycotoxins (FBs, DON, ZEN, AFB_1_ and OTA) with varying degrees of contamination in maize kernels were also analyzed in Northern Serbia [13]. Mycotoxins not only contaminate maize, but also wheat, soybeans, feed and so on. The contamination of AFBs and OTA in imported and Lebanese wheat and its products also pose a serious threat to the health of consumers [14]. In addition, multiple different mycotoxins were observed simultaneously in feed samples [15]. Fish feed samples from several fish farms and fish feed factories in the Lake Victoria basin were contaminated with various mycotoxins [16]. Kernel samples from Rabat and Serine markets in Morocco were also contaminated with a variety of mycotoxins, among which the average concentration of FBs in maize samples was up to 1930 μg/kg [17]. Samples of feed and feed materials including pig, cattle and poultry feeds were contaminated with at least one mycotoxin, including AFB_1_, ZEN and FBs [18,19]. Mycotoxin contamination was affected by a variety of factors, including geographic location, climatic conditions, varietal differences and cultivation methods, but climate was a key factor [20]. Among several environmental variables studied, it was found that temperature and precipitation were the most influential factors for mycotoxins [21,22].

In our previous study, we have been continuously monitoring the contamination of FBs in maize grains in different regions of China for 10 years since 2011. Affected by various factors such as climate conditions, various types, and cultivation methods, the contamination levels of FBs are different and the contamination situation was not optimistic. In order to better monitor and control the contamination of mycotoxins in the two major maize-producing areas in China and ensure food safety and people’s health, we used ultra-performance liquid chromatography–mass spectrometry (UPLC–MS/MS) to monitor the mycotoxins content in 426 maize kernels from the northeast and northwest China.

## 2. Results and Discussion

### 2.1. Occurrence of Mycotoxins in North China

**Figure 1:**
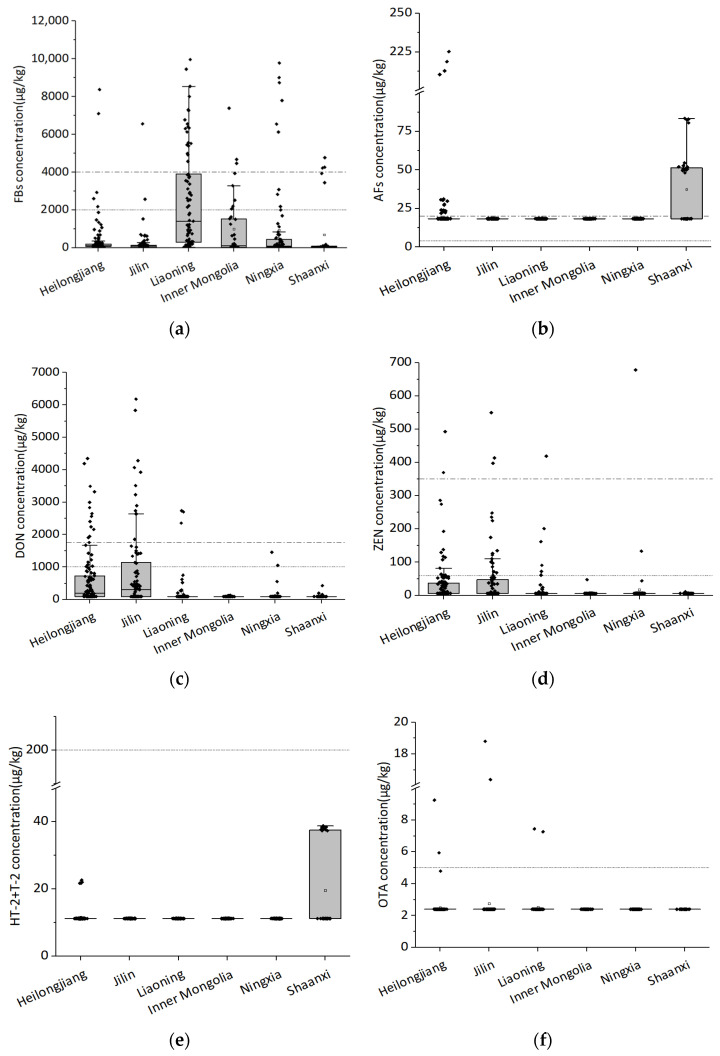
The contamination levels of mycotoxins in maize samples from six provinces in China. (**a**) The contamination levels of FBs in maize samples from six provinces in China. (**b**) The contamination levels of AFs in maize samples from six provinces in China. (**c**) The contamination levels of DON in maize samples from six provinces in China. (**d**) The contamination levels of ZEN in maize samples from six provinces in China. (**e**) The contamination levels of HT-2+T-2 in maize samples from six provinces in China. (**f**) The contamination levels of OTA in maize samples from six provinces in China.

In this study, 11 mycotoxins were analyzed in 426 maize kernel samples from six provinces and autonomous regions in northwest and northeast China. The contamination levels of mycotoxins in maize kernels are shown in Figure 1. The results show that the contamination of maize seeds with mycotoxins vary widely in different regions of China, among which FBs and DON were most serious. The positive rate of FBs was 89.7%, with the average contamination level of 937 μg/kg. According to the results, the average concentration of FBs in Liaoning province was higher than that in other provinces. Similarly, by comparing the contamination status of maize kernel samples from some regions in the northeast and northwest China in 2014, it can be seen that Liaoning province was also the most contaminated [10]. By comparing the climatic conditions in Table 1, the average temperature of Liaoning province was higher than that of other provinces, which may lead to a relatively high level of FB contamination, which was consistent with the research results of Li and Wei, indicating that air temperature was closely related to FB contamination [10,23,24]. 

**Figure 2:**
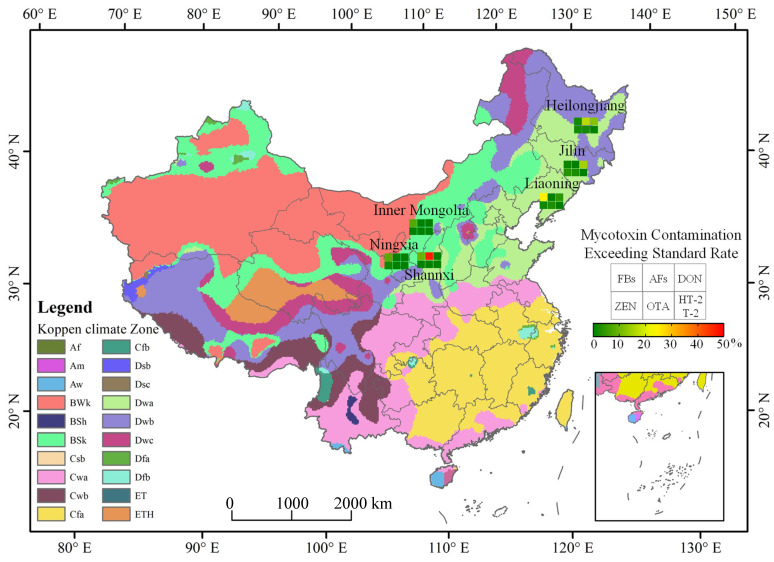
The Köppen–Geiger Climate Classification and mycotoxins occurrence in China.

In terms of AFs (AFB_1_ + AFB_2_ + AFG_1_ + AFG_2_) contamination, the average contamination level of AFs was 22 μg/kg, with an overall positive rate of 8.0%. Compared with other provinces, maize kernels in Shaanxi province were the most seriously contaminated by AFs. In terms of DON, the average contamination level of DON was 431 μg/kg, with an overall positive rate of 33.1%, while for ZEN, it was 27 μg/kg and 23%. In six provinces, for both DON and ZEN, Jilin and Heilongjiang province were the most contaminated. As for HT-2 and T-2, Shaanxi province was the most contaminated. The contamination was below the limit of quantification in maize samples from other regions. Except for a few samples, the content of OTA in the tested maize samples was below the limit of quantification. There were also low-cost and rapid methods for analyzing the OTA content in maize [25], but a rapid method to analyze several mycotoxins at the same time remains to be discovered. Compared with the northwest region, the mycotoxin contamination in the maize samples from the northeast region was more serious, which may be related to the local climate conditions. According to the temperature data and the precipitation value of different regions in Table 1, compared with the northwest region, the monthly average temperature in northeast China was higher than that in northwest China, which was consistent with the relationship between mycotoxin contamination monitoring and environmental factors in other countries. According to the Köppen–Geiger climate classification map of the six tested regions and mycotoxin occurrence in Figure 2, it can be seen that the maize growing areas in Inner Mongolia and Ningxia belong to the BWk (Dry Tropical Climate) and BSk climate types (Dry Midlatitude Climates). The average temperature in these regions during the maize silking period was 32.5 °C and 19.0 °C. Judging from our monitoring, the excess rate of mycotoxins was low with no safety risk. The maize growing areas in Heilongjiang, Jilin, Liaoning and Shaanxi provinces belong to the Dwa climate type (Temperate monsoon climate) mainly. The average temperature of the climate types during the maize silking period was 23.1 °C, with a higher precipitation. In these places, we monitored more maize samples that exceed the standard. However, it was generally relatively safe, with only a few toxins exceeding the standard. These data suggest that mycotoxin contamination status was closely related to temperature and water activity, which was also consistent with previous reports. The FB contamination was related to the temperature and water activity. The FBs synthesis ability of *F. verticillioides* at 25 °C with 0.99 aw was higher than at 35 °C with a low water activity [26]. It was reported that AFB_1_ also has an environmental response phenomenon. Under the conditions of 25–33 °C and 0.90–0.99 aw, the yield of AFB_1_ was higher, but not detected at 20 °C with Aw ≤ 0.90 and 37 °C with Aw ≥ 0.96 [27]. The contamination of AFs was analyzed in maize samples under climate conditions in Pakistan and it was found that the AF content of maize samples was higher in months with higher average temperature [28]. The global occurrence of mycotoxins such as AFB_1_, FBs and ZEN in feed and feed material samples were analyzed from different countries and regions in the past 10 years, and it found that the temperature of maize silking stage in China’s maize core growing regions in 2017 was higher than that in 2013–2016. As a result, the concentrations of FBs and AFB_1_ in maize in 2017 were significantly higher than those in previous years [19]. In addition to the influence of temperature, humidity and other climatic conditions, pre-harvest practices were significantly correlated with crop mycotoxins contamination [29], and poor pre-harvest agricultural practices can lead to increased mycotoxin contamination of crops [30]. Affected by various environmental factors, mycotoxins may vary according to global geographical differences [31].

### 2.2. Contamination Levels of Mycotoxins in North China

Since these mycotoxins endanger human health and safety, various organizations have established limit standards for each mycotoxin [32,33,34,35,36], as shown in Table 2. The contamination levels of mycotoxins in maize kernel samples were compared with their limits, as shown in Table 3. Among 426 maize kernel samples tested, five maize samples contained more than 2000 μg/kg levels of FBs, accounting for 4.4% of the total samples. Among them, two samples exceeded 4000 μg/kg, which exceeded the EU limit, accounting for 1.8% of the total samples. On the whole, except for Liaoning province, most maize kernel samples contained safe levels of FBs. The content of all the samples tested were higher than the limit standard of EU, but the AF concentration of only some maize samples was slightly higher than FDA limit standard. For other mycotoxins, although some samples exceeded their own limits, almost all were at safe levels. Nonetheless, the status of mycotoxins in maize was potentially dangerous. Therefore, in order to further prevent and control the harm of mycotoxins to human health, it was still necessary to pay more attention to the quality of maize samples from different regions and the cultivation methods in the planting process. We also continuously monitor the occurrence of mycotoxins in maize. Food safety supervision should be coordinated by various channels and organizations to further improve supervision efficiency [37]. The task of food safety supervision was still arduous.

## 3. Conclusions

Herein, we established a method for the simultaneous analysis of 11 mycotoxins residues in maize kernel samples, and the recoveries were in compliance with the relevant regulations of residue limits. The average value, the positive rate, the proportion exceeding the maximum limit standard of FBs, DON, AFs, ZEN, OTA and T-2 + HT-2 was 937, 431, 22, 27, 2 and 12 μg/kg; 89.7%, 33.1%, 8.0%, 23%, 1.6%, 3.3%; 8.0%, 7.0%, 8.0%, 1.6%, 1.4% and 0.0%. At the same time, based on the contamination of 426 maize samples, we estimate that FBs and DON contamination in maize kernels was more serious, 8% of the samples have exceeded the maximum limit standard. However, it was generally lower than its limit standard and even needs further monitoring. Contamination levels of mycotoxins vary in different regions. In general, the average value of mycotoxins differs significantly between regions only in adverse weather conditions. Overall, we found that the most serious FBs contamination was in Liaoning province, and the contamination of AFs and HT-2 + T-2 in maize grain samples from Shaanxi province was the most serious. For DON and ZEN, Heilongjiang and Jilin province were the most seriously contaminated. Almost none of the tested maize samples were contaminated with OTA. Comparison of the Köppen–Geiger climate classification map, it can be concluded that mycotoxin contamination is more serious in humid regions.

## 4. Materials and Methods

### 4.1. Samples

The samples used in this experiment were 426 healthy maize kernel samples collected from northwest China (Shaanxi Province, Ningxia Region, Inner Mongolia Region) and northeast China (Heilongjiang Province, Jilin Province, Liaoning Province), provided by Maize Industry Technology System Test Station. The cultivation methods and drug use of the experimental fields were based on the management habits of local farmers. The temperature conditions of maize in the silking period in the studied provinces and regions are summarized in Table 1. Our maize seeds were sown in June every year and harvested in October of that year from 2018 to 2020. There was no mold on the surface of the maize kernels. After all samples were air-dried, each maize sample was randomly weighed at 200 g and added to the mill for grinding, and each maize sample was crushed through a 500 μm mesh sieve, and wrefrigerated at −20 °C until mycotoxins analysis.

### 4.2. Chemical Standards and Reagents

Standards of FB_1__,_ FB_2__,_ AFB_1__,_ AFB_2__,_ AFG_1__,_ AFG_2__,_ DON, ZEN, OTA, T-2, HT-2 were obtained from Pribolab Co. (Qingdao, China). HPLC-grade acetonitrile, methanol, and ammonium acetate were supplied by Thermo Fisher Scientific (Waltham, MA, USA), respectively, and HPLC-grade formic acid were purchased from aladdin Co. (Shanghai, China), which were used for sample preparation and mobile phase. Analytical grade acetonitrile was purchased from kermel Co. (Tianjin, China). Ultrapure water was prepared in all analytical steps using a Barnstead Lab Tower EDI water purification system (Thermo Fisher Scientific, Waltham, MA, USA).

### 4.3. Sample Pretreatment

Samples were processed according to the determination method of mycotoxins in main kernels as specified in the food industry standard of China [31], 5 g maize kernel samples that had been ground and crushed were accurately weighed and put into a 50 mL centrifuge tube. 20 mL extraction solution, i.e., acetonitrile: water: acetic acid (70:29:1, *v/v/v*) was added. After swirling for 30 min, centrifuged at 6000 r/min for 10 min, 0.5 mL supernatant was obtained into a 1.5 mL centrifuge tube and 0.5 mL ultra-pure water was added. Then, the solution was vortex mixed and centrifuged at 10,000 r/min for 10 min, and the supernatant was filtered through a 0.22 μm poly tetra luoroethylene filter for subsequent analysis.

### 4.4. UPLC–MS/MS Analysis

Liquid chromatography conditions: A Bonshell C_18_ (50 mm × 2.1 mm, 2.7 µm particle size) analytical column with a C_18_ security guard cartridge from Agela Technologies was employed for the chromatographic separation. The flow rate was 0.3 mL/min, with the column temperature of 35 °C. The injection volume was 2 μL. The mobile phase was an aqueous solution of 5 mmol/L ammonium acetate containing 0.1% formic acid (phase A) and methanol (phase B). Gradient elution was started with 5% phase B. Then, phase B was linearly increased to 95% within 5 min and kept constant for 1 min. Finally, phase B was decreased to 10% in 0.1 min and equilibrated for 2 min.

Mass spectrometry conditions: carrier gas: N2 with purity ≥99.999%; curtain gas: 30 psi; collision gas: 7 psi; ionization temperature: 550 °C; atomized gas: 50 psi; auxiliary gas: 50 psi; multiple ion reaction monitoring mode. The mass spectrum parameters of 11 mycotoxins are shown in Table 4.

In order to analyze many mycotoxins in maize kernel with high throughput, the analysis conditions of UPLC–MS/MS were optimized. The standard curve was constructed with seven horizontal curve points of 1, 5, 10, 50, 100, 500 and 1000 μg/kg prepared by sample blank extract solution. Standard curves were drawn with the concentrations of the above 11 mycotoxins as the horizontal coordinate and the peak area as the vertical coordinate. As can be seen from Table 5, these mycotoxins have a good linear relationship within their respective linear range, with coefficient of determination (R^2^) > 0.99. Limits of detection (LOD) and limits of quantification (LOQ) are also shown in Table 5 based on signal-to-noise ratios (S/N) of 3 and 10, respectively. In order to verify the accuracy and precision of the method, a recovery experiment was conducted by adding mixed matrix standard stock solutions to the concentration of blank samples at 20, 500 and 1600 μg/kg into blank samples with five replicates, which are shown in Table 6.

### 4.5. Koppen–Geiger Climate Classification Map of China

We drew the Koppen–Geiger climate classification map of China with reference to the Köppen climate classification map [38]. The Koppen–Geiger climate map was based on a large dataset of monthly precipitation and air temperature over many years. The map was divided into tropical (Type A), arid (Type B), temperate (Type C), cold (Type D) and Polar (Type E) five primary climate types. On the basis of this figure, the proportion of mycotoxins exceeding the maximum limit standard in six regions of China was added.

## Figures and Tables

**Table 1:** Climate characteristics of six provinces in China during maize field experiments.

Province	Mean Maximum Temperature (°C)	Mean Minimum Temperature (°C)	Mean Temperature (°C)	Mean Precipitation Value (mm)
Heilongjiang	27.6	19.1	23.3	164.3
Jilin	28.2	19.3	23.8	93.8
Liaoning	29.9	21.0	25.4	98.3
Inner Mongolia	29.1	19.4	24.3	100.5
Ningxia	29.2	18.9	24.1	41.5
Shaanxi	27.7	19.0	23.3	150.2

**Table 2:** Limits of mycotoxins (µg/kg) in maize by different organizations and country.

Mycotoxin	Organizations and Country		
	China	FDA	EU
FBs	-	2000	4000
AFB_1_ + AFB_2_ + AFG_1_ + AFG_2_	-	20	4
DON	1000	-	1750
ZEN	60	-	350
OTA	5	-	5
T-2 + HT-2	-	-	200

**Table 3:** Distribution (%) of mycotoxins contamination levels in maize samples from six provinces in China.

Mycotoxin	Concentration (µg/kg)	Province					
		Heilongjiang	Jilin	Liaoning	Inner Mongolia	Ningxia	Shaanxi
**FBs**	0 < x < 2000	95.6%	97.7%	57.1%	80.0%	86.8%	84.4%
	2000 < x < 4000	2.6%	1.1%	18.2%	12.5%	5.3%	6.3%
	x > 4000	1.8%	1.1%	24.7%	7.5%	7.9%	9.4%
**AFs**	0 < x < 4	0.0%	0.0%	0.0%	0.0%	0.0%	0.0%
	4 < x < 20	83.3%	100.0%	100.0%	100.0%	100.0%	53.1%
	x > 20	16.7%	0.0%	0.0%	0.0%	0.0%	46.9%
**DON**	0 < x < 1000	80.7%	73.6%	96.1%	100.0%	97.4%	100.0%
	1000 < x < 1750	7.0%	11.5%	0.0%	0.0%	2.6%	0.0%
	x > 1750	12.3%	14.9%	3.9%	0.0%	0.0%	0.0%
**ZEN**	0 < x < 60	87.7%	79.3%	92.2%	100.0%	97.4%	100.0%
	60 < x < 350	10.5%	17.2%	6.5%	0.0%	1.3%	0.0%
	x > 350	1.8%	3.4%	1.3%	0.0%	1.3%	0.0%
**OTA**	0 < x < 5	98.2%	97.7%	97.4%	100.0%	100.0%	100.0%
	x > 5	1.8%	2.3%	2.6%	0.0%	0.0%	0.0%
**HT-2 + T-2**	0 < x < 200	100.0%	100.0%	100.0%	100.0%	100.0%	100.0%
	x > 200	0.0%	0.0%	0.0%	0.0%	0.0%	0.0%

**Table 4:** UPLC–MS/MS parameters of 11 mycotoxins.

Mycotoxins	Precursor Ion (*m/z*)	Product Ion (*m/z*)	Residence Time (min)	DP (V)	CE (eV)	Ionization Mode (ESI)	Ionspray Voltage (V)
FB_1_	722.4	334.3 *,352.3	4.17	220	45,45	+	5500
FB_2_	706.5	336.3 *,318.3	4.61	112	45,45	+	5500
AFB_1_	313.2	285.1 *,241.1	3.59	121	22,38	+	5500
AFB_2_	315.2	287.1 *,259.1	3.48	124	24,30	+	5500
AFG_1_	329.2	311.1 *,243.1	3.33	120	20,25	+	5500
AFG_2_	331.2	313.1 *,245	3.19	122	33,40	+	5500
OTA	404.1	239 *,358	4.58	120	25,10	+	5500
HT-2	442.2	215.1 *,263.1	4.02	75	10,10	+	5500
T-2	484.3	305 *,185.1	4.30	80	18,30	+	5500
DON	297.1	249.1 *,203.1	1.74	180	15,19	+	5500
ZEN	317.1	175 *,130.8	4.57	95	25,33	-	4500

Note: DP, declustering potential; CE, collision energy; *—quantitative ion.

**Table 5:** Linearity and sensitivity of the applied LC/MS method.

Mycotoxin	LOD (μg/kg)	LOQ (μg/kg)	Linearity Equation	R^2^
FB_1_	0.8	2.4	Y = 2048.6X + 5164.4	0.9997
FB_2_	0.3	0.9	Y = 5920.2X + 7589.1	0.9998
DON	3.9	11.9	Y = 233.08X + 350.97	0.9997
AFB_1_	0.2	0.5	Y = 12923X + 25907	0.9998
AFB_2_	0.3	0.8	Y = 9854.5X + 18439	0.9998
AFG_1_	0.3	0.8	Y = 14893X + 36370	0.9997
AFG_2_	0.1	0.2	Y = 22657X + 84711	0.9995
OTA	0.1	0.3	Y = 12886X − 10112	0.9999
T-2	0.1	0.3	Y = 7452.9X − 16843	0.9997
HT-2	0.4	1.1	Y = 838X + 2102.7	0.9997
ZEN	0.3	0.8	Y = 3840.1X + 4480.3	0.9999

Note: R^2^, coefficient of determination; LOD, limit of detection; LOQ, limit of quantitation.

**Table 6:** Mean recoveries and relative standard deviations of mycotoxins in spiked maize samples.

Mycotoxin	Recovery (%)	RSD (%)	Recovery (%)	RSD (%)	Recovery (%)	RSD (%)
	20 μg/kg		500 μg/kg		1600 μg/kg	
FB_1_	87	9.2	83	3.7	75	4.2
FB_2_	81	4.4	82	7.2	74	6.3
DON	84	8.4	95	8.5	73	6.2
AFB_1_	78	7.1	84	8.0	89	9.1
AFB_2_	85	8.1	86	4.9	85	4.7
AFG_1_	92	6.6	95	4.8	88	5.9
AFG_2_	78	5.3	89	3.8	97	7.7
OTA	72	3.5	83	4.4	75	2.9
T-2	82	8.6	89	5.3	82	8.4
HT-2	77	7.2	91	9.0	85	4.7
ZEN	78	7.3	83	3.0	76	4.3

Note: RSD-Relative standard deviation.

## Data Availability

The data presented in this study are available in this article.
